# No association between atopic outcomes and type of pertussis vaccine given in children born on the Isle of Wight 2001-2002

**DOI:** 10.1016/j.jaip.2016.06.005

**Published:** 2016

**Authors:** Carina Venter, Julia Stowe, Nick J. Andrews, Elizabeth Miller, Paul J. Turner

**Affiliations:** a The David Hide Asthma and Allergy Research Centre, St Mary's Hospital, Newport, Isle of Wight, United Kingdom; b School of Health Sciences and Social Work, University of Portsmouth, Portsmouth, United Kingdom; c Immunisation, Hepatitis and Blood Safety Department, Public Health England, London, United Kingdom; d The Section of Paediatrics (Allergy and Infectious Diseases) and MRC and Asthma UK Centre in Allergic Mechanisms of Asthma, Imperial College London, London, United Kingdom

To the Editor:

**Clinical Implications**

• The increase in food allergy prevalence has coincided with changes in pertussis vaccine used for infant immunization. In a prospective birth cohort study, we report no association between atopic outcomes and type of vaccine used.

Pertussis is typically included in most infant vaccination schedules. The development of atopic sensitization occurs early in infancy; thus, the infant vaccine schedule may have an impact on atopic outcomes. A link between pertussis immunization and risk of atopic disease was first suggested for whole-cell pertussis (wP),^1^, ^2^ although the published data strongly suggest that these concerns are unwarranted.^3^, ^4^, ^5^

More recently, many countries have switched from wP to acellular pertussis (aP), a measure instituted to reduce the relatively higher rate of adverse events associated with wP immunization. The aP vaccine drives a T_H_2-like immune response in contrast to the wP vaccine,^6^, ^7^ which might predispose to atopic disease. Public health bodies in the United States, Australia, and Europe have noted that in some countries, the increase in prevalence of food allergy in the last few decades has coincided with the switch from wP to aP. There are no published studies assessing how the risk of atopic disease is affected by type of pertussis immunization, although data suggest that IgE production following a booster with aP appears to be specific for pertussis-related antigens and not for food or environmental allergens.^8^

In the United Kingdom, the switch from wP to aP occurred in 2004. However, in 1999-2001, a shortage in the supply of wP resulted in the release of aP to meet demand. This period coincided with the establishment of a birth cohort study to assess the epidemiology of atopic disease. As a result, infants included in the birth cohort received a mix of wP and aP almost at random, depending on the supply of the particular vaccine, avoiding potential biases due to secular trends in the risk of developing atopic disease.

The Food Allergy and Intolerance Research (FAIR) birth cohort included all 1063 babies born on the Isle of Wight (UK) between September 2001 and August 2002. A total of 969 (91%) parents consented to allergy assessments being performed on their child at age 1, 2, 3, and 10 years.^9^ Symptoms of allergic disease were assessed using validated questionnaires,^4^ in combination with parent interview. Information on family history and history of allergic disease was obtained by questionnaire. Children underwent skin prick tests (SPTs) to a standard panel of predefined food (milk, egg, wheat, peanut, sesame, and cod fish) and aeroallergens (house dust mite *Dermatophagoides pteronyssinus*, cat, and grass). A positive SPT result was defined as a mean wheal size of 3 mm or more. Children with a history of possible allergic reaction and/or positive SPT result to a food were invited to undergo oral food challenge (double-blind placebo-controlled food challenge).^9^

We obtained data relating to vaccine status (type of vaccine, date given) of children included in the FAIR cohort from a centralized register held by Public Health England. A selection of paper child-health records was cross-referenced with this data set to confirm data integrity. Analysis was limited to children who received their first dose of pertussis vaccine between the age of 6 weeks and 18 weeks and in whom the type of vaccine was recorded. To compare the risks of allergy according to aP and wP exposure, we performed multivariable binomial regression to derive crude relative risks and relative risks adjusted for potential confounders of family history of asthma/hay fever, breast-feeding, and sex. Ethical approval for the study was obtained from the South Central – Southampton B Research Ethics Committee (REF 10/H0504/11), with data linkage under separate approval granted to Public Health England.

Details regarding the first pertussis vaccine administered were available for 906 children: 71 received their dose after age 18 weeks, and were therefore excluded, while in 16 children, we were unable to determine the type of pertussis vaccine administered. Thus, 819 children were included in the initial analysis, of whom 224 (27%) received their first vaccine dose containing aP (and the remaining 595 [73%] wP). No significant associations were identified between any outcome measure and type of pertussis vaccine used for the first infant vaccine (Table I).

To assess whether receipt of any dose of aP was associated with a change in risk, we performed a further analysis in 701 children who received 3 doses of pertussis according to the immunization schedule and in whom data regarding the type of vaccine (aP vs wP) were available. A total of 343 (48.9%) received at least 1 dose of aP, whereas 351 (50.1%) received wP for all 3 doses. No significant associations were identified between any outcome measure and administration of at least 1 dose of aP in the first year of life (Table II). We also assessed for any effect on outcome by dosing trend; that is, which vaccine (aP or wP) was used for each dose: no significant associations were identified (Table II).

In summary, we did not identify any evidence for an association between type of pertussis vaccine given and allergic/atopic outcomes in this cohort. The strengths of this study include the almost random allocation of vaccine (wP vs aP), prospective data collection, use of objective assessments, and high rate of follow-up; however, our analysis is limited by the size of this cohort and we cannot, therefore, exclude a more subtle effect of acellular pertussis on subsequent atopy.

## Figures and Tables

**Table I:** RRs of atopic outcomes in those receiving a first dose of aP vs wP, adjusted for potential confounders

Outcome	First dose aP, n/N∗ (%)	First dose wC, n/N∗ (%)	RR (95% CI)	Fisher exact *P* value	Adjusted RR†	*P* value
IgE-mediated food allergy, ever	5/223 (2.2%)	19/591 (3.2%)	0.70 (0.26-1.84)	.64	0.78 (0.29-2.07)	.62
Positive SPT result to food, ever	5/174 (2.9%)	30/465 (6.5%)	0.45 (0.18-1.13)	.08	0.46 (0.18-1.17)	.10
Asthma, by age 3 y	22/204 (10.8%)	54/560 (9.6%)	1.12 (0.70-1.79)	.68	1.16 (0.71-1.87)	.55
Asthma, by age 10 y	28/112 (25.0%)	60/336 (17.9%)	1.40 (0.94-2.08)	.10	1.15 (0.73-1.81)	.55
Eczema, at 6 mo	84/217 (38.7%)	230/568 (40.4%)	0.96 (0.79-1.16)	.68	0.84 (0.77-1.15)	.54
Eczema, at 1 y	35/212 (16.5%)	112/564 (19.95)	0.83 (0.59-1.17)	.31	0.84 (0.59-1.20)	.35
Eczema at 3 y	40/202 (19.8%)	103/556 (18.5%)	1.07 (0.77-1.48)	.68	1.06 (0.76-1.47)	.74
Allergic rhinitis, by age 10 y	54/191 (28.2%)	142/526 (27.0%)	1.05 (0.80-1.37)	.78	1.05 (0.81-1.38)	.70
Any positive SPT result to aeroallergen, ever	49/202 (24.3%)	99/538 (18.4%)	1.32 (0.97-1.78)	.08	1.24 (0.90-1.69)	.19
Sensitized to HDM, ever	30/118 (25.4%)	56/320 (17.5%)	1.45 (0.98-2.14)	.08	1.30 (0.86-1.98)	.22
Any atopy	51/203 (25.1%)	106/538 (19.7%)	1.27 (0.95-1.71)	.11	1.19 (0.88-1.62)	.25

*HDM*, House dust mite; *RR*, relative risk.

The 95% CIs for the RRs show the precision of estimates for the different outcomes.

∗ Where denominators do not add up to 819, this is due to missing data.

† Adjusted for family history of asthma/hay fever, breast-feeding, and sex.

**Table II:** RRs of atopic outcomes in those receiving any dose of aP vs none (ie, wP used for all 3 immunizations), adjusted for potential confounders

Outcome	Any aP, n/N∗ (%)	No aP (ie, wP used for all 3 doses), n/N∗ (%)	RR (95% CI) (any aP vs none)	Fisher exact *P* value	Adjusted RR† (any aP vs none)	*P* value	Trend per dose (adjusted RR† per aP dose)
IgE-mediated food allergy, ever	10/340 (2.9%)	8/356 (2.2%)	1.30 (0.52-3.28)	.64	1.16 (0.46-2.97)	.75	1.17 (0.75-1.80)
Positive SPT result to food, ever	13/274 (4.7%)	16/297 (5.4%)	0.88 (0.43-1.79)	.85	0.76 (0.37-1.58)	.47	0.87 (0.59-1.27)
Asthma, by age 3 y	37/315 (11.7%)	32/341 (9.4%)	1.25 (0.80-1.96)	.35	1.13 (0.71-1.80)	.62	1.12 (0.90-1.38)
Asthma, by age 10 y	41/178 (23.0%)	37/220 (16.8%)	1.37 (0.92-2.04)	.13	1.21 (0.79-1.87)	.39	1.04 (0.84-1.29)
Eczema, at 6 mo	125/328 (38.1%)	145/346 (41.9%)	0.91 (0.76-1.09)	.35	0.88 (0.73-1.05)	.17	0.94 (0.86-1.04)
Eczema, at 1 y	56/325 (17.2%)	73/345 (21.1%)	0.81 (0.60-1.11)	.20	0.82 (0.59-1.12)	.21	0.91 (0.78-1.08)
Eczema at 3 y	56/313 (17.9%)	70/338 (20.7%)	0.86 (0.63-1.18)	.37	0.85 (0.62-1.17)	.32	0.95 (0.81-1.12)
Allergic rhinitis, by age 10 y	80/301 (26.6%)	98/322 (30.4%)	0.87 (0.68-1.12)	.33	0.88 (0.68-1.13)	.31	0.95 (0.84-1.08)
Any positive SPT result to aeroallergen, ever	73/311 (23.5%)	60/332 (18.1%)	1.30 (0.96-1.76)	.10	1.17 (0.86-1.60)	.31	1.11 (0.96-1.28)
Sensitized to HDM, ever	43/187 (23.0%)	36/217 (16.6%)	1.39 (0.92-2.06)	.13	1.14 (0.75-1.73)	.54	1.13 (0.94-1.36)
Any atopy	77/312 (24.7%)	64/332 (19.3%)	1.28 (0.95-1.72)	.11	1.16 (0.86-1.57)	.32	1.09 (0.95-1.26)

*HDM*, House dust mite; RR, relative risk.

We also assessed for any effect on outcome by dosing trend, ie, which vaccine (aP or wP) was used for each dose. The 95% CIs for the RRs show the precision of estimates for the different outcomes.

∗ Where denominators do not add up to 701, this is due to missing data.

† Adjusted for family history of asthma/hay fever, breast-feeding, and sex.
